# Classifier algorithms to characterize various stages of Alzheimer’s disease based on analysis of circulating brain‐enriched and inflammation‐associated microRNAs

**DOI:** 10.1002/alz.095519

**Published:** 2025-01-09

**Authors:** Arzu Kunwar, Kenny K Ablordeppey, Jacob Goldman, Alidad Mireskandari, Gyanendra Kumar, Michael Kiefer, David A Wolk, Kira S. Sheinerman, Samuil R. Umansky

**Affiliations:** ^1^ DiamiR Biosciences, New Haven, CT USA; ^2^ Department of Neurology, University of Pennsylvania, Philadelphia, PA USA

## Abstract

**Background:**

Minimally invasive diagnostic tools are crucial to characterize heterogeneous mild cognitive impairment (MCI), pre‐MCI, and Alzheimer’s disease (AD) cohorts. Our approach is based on targeted selection and quantitative analysis of microRNAs enriched in the brain and detectable in blood plasma. Here, we report the comparative analysis of 24 microRNAs comprising CogniMIR panel in 200 plasma samples with our Gen1 and Gen2 software algorithms towards distinguishing cognitively unimpaired (CU), MCI and AD cohorts.

**Method:**

The expression of 24 brain‐enriched and inflammation‐associated microRNAs were analyzed in 200 plasma samples collected at the Univ. of Pennsylvania’s ADRC from 76 CU, 85 MCI, and 39 AD study participants. RNA from plasma samples was isolated using the MagMAX mirVana kit (ThermoFisher). microRNA concentrations were measured by qPCR using custom plates (Qiagen) pre‐plated with lyophilized miRCURY LNA‐based primers. Performance of classifier algorithms in differentiating clinically distinct groups was evaluated using Gen1 (self‐validation) and Gen2 (five‐fold cross validation) algorithms.

**Result:**

AUC generated with Gen1 for differentiation of CU Aβ‐/APOE4‐ vs AD APOE4+ was 91%, while the mean of five‐fold cross validation AUC for the same cohort was 85%. Similarly, the self‐validation AUC for CU Aβ‐/APOE4‐ vs MCI Aβ+/APOE4+ was 82%, while the five‐fold cross validation AUC was 77%. Further, the self‐validation AUC for CU Aβ‐/APOE4‐ vs CU Aβ+/APOE4+ was 85%, whereas the AUC generated with the five‐fold cross validation for the same groups was 61%. These analyses demonstrate higher self‐validation performance metrics as compared to the five‐fold cross validation metrics. It is likely that self‐validation (Gen1), which utilizes the full sample set for the analysis, provides an overestimation of the classifier’s performance. Whereas, in the five‐fold validation (Gen2) 20% of samples are held back during the classifier training step and used for testing evaluation; this process is repeated five times with different withheld subsets, and the performance results are averaged out.

**Conclusion:**

DiamiR’s Gen2 classifier algorithm offers a more reliable estimate of generalized performance compared to our Gen1 classifier. The data generated in this study further support the development of assays based on brain‐enriched and inflammation‐associated microRNAs detectable in the blood. A larger study is ongoing.

